# Domain Mapping and Deep Learning from Multiple MRI Clinical Datasets for Prediction of Molecular Subtypes in Low Grade Gliomas

**DOI:** 10.3390/brainsci10070463

**Published:** 2020-07-18

**Authors:** Muhaddisa Barat Ali, Irene Yu-Hua Gu, Mitchel S. Berger, Johan Pallud, Derek Southwell, Georg Widhalm, Alexandre Roux, Tomás Gomez Vecchio, Asgeir Store Jakola

**Affiliations:** 1 Department of Electrical Engineering, Chalmers University of Technology, 41296 Gothenburg, Sweden; barat@chalmers.se (M.B.A.); irenegu@chalmers.se (I.Y.-H.G.); 2 Department of Neurological Surgery, University of California San Fransisco, San Francisco, CA 94143-0112, USA; Mitchel.Berger@ucsf.edu (M.S.B.); dereksouthwell@gmail.com (D.S.); 3 Department of Neurosurgery, GHU Paris—Sainte-Anne Hospital, University of Paris, F-75014 Paris, France; j.pallud@ghu-paris.fr (J.P.); alexandre.roux@neurochirurgie.fr (A.R.); 4 Department of Neurosurgery, University Hospital of Vienna, 1090 Vienna, Austria; georg.widhalm@meduniwien.ac.at; 5 Department of Clinical Neurosciences, Institution of Neuroscience and Physiology, Sahlgrenska Academy, 41345 Gothenburg, Sweden; tomas.gomez.vecchio@gu.se

**Keywords:** CycleGAN, 1p/19q codeletion, IDH genotype, domain mapping, brain tumor, deep learning

## Abstract

Brain tumors, such as low grade gliomas (LGG), are molecularly classified which require the surgical collection of tissue samples. The pre-surgical or non-operative identification of LGG molecular type could improve patient counseling and treatment decisions. However, radiographic approaches to LGG molecular classification are currently lacking, as clinicians are unable to reliably predict LGG molecular type using magnetic resonance imaging (MRI) studies. Machine learning approaches may improve the prediction of LGG molecular classification through MRI, however, the development of these techniques requires large annotated data sets. Merging clinical data from different hospitals to increase case numbers is needed, but the use of different scanners and settings can affect the results and simply combining them into a large dataset often have a significant negative impact on performance. This calls for efficient domain adaption methods. Despite some previous studies on domain adaptations, mapping MR images from different datasets to a common domain without affecting subtitle molecular-biomarker information has not been reported yet. In this paper, we propose an effective domain adaptation method based on Cycle Generative Adversarial Network (CycleGAN). The dataset is further enlarged by augmenting more MRIs using another GAN approach. Further, to tackle the issue of brain tumor segmentation that requires time and anatomical expertise to put exact boundary around the tumor, we have used a tight bounding box as a strategy. Finally, an efficient deep feature learning method, multi-stream convolutional autoencoder (CAE) and feature fusion, is proposed for the prediction of molecular subtypes (1p/19q-codeletion and IDH mutation). The experiments were conducted on a total of 161 patients consisting of FLAIR and T1 weighted with contrast enhanced (T1ce) MRIs from two different institutions in the USA and France. The proposed scheme is shown to achieve the test accuracy of 74.81% on 1p/19q codeletion and 81.19% on IDH mutation, with marked improvement over the results obtained without domain mapping. This approach is also shown to have comparable performance to several state-of-the-art methods.

## 1. Introduction

Diffuse gliomas are the most common type of cancer originating from the brain. Based on histological and molecular features, they have been graded by the World Health Organization from grade II-IV and classified as either astrocytomas and oligodendrogliomas [1]. Diffuse low grade gliomas (WHO grade II) can consequently be classified on the basis of IDH mutation and 1p/19q codeletion and this has a major impact on prognosis and response to therapy [2]. Oligodendrogliomas contain IDH mutation and 1p19q codeletion, while astrocytomas have no codeletion and are further subclassified if they are IDH mutated or not. IDH wild-type gliomas are molecularly similar to GBMs and have poor prognosis. Low grade gliomas (LGGs) tend to present with seizures and typically involve the frontal lobes, and these tumors usually do not show significant contrast enhancement while some of the oligodendrogliomas contain radiographically detectable calcification. The molecular information would be of practical value since oligodendrogliomas harbor better prognosis than the other LGG subtypes and also seem to be more sensitive to oncological treatment [3,4]. This molecular information requires a tissue diagnosis, but recently several advanced machine learning techniques have been shown to predict molecular subtypes in gliomas based upon preoperative imaging [5,6,7,8]. Non-invasive diagnostic tools are attractive in identification since it may assist in prognostication and would significantly enhance patient counseling and shared decision making. However, major challenges still remain before putting these tools into clinical use.

### Related Work

Machine learning methods for classifying gliomas are either based on hand-crafted features or automatic learning of features. Kang et al. [9] introduced a method using diffusion weighted MRIs based on histogram analysis of diffusion coefficient maps over the entire volume of tumor for glioma grading. Zhou et al. [7] used histogram, shape and texture features combined with age information to a random forest algorithm for IDH mutation and 1p/19q codeletion prediction. Han et al. [5] performed an analysis to generate radiomics signature by extracting 647 MRI based features for predicting 1p/19q codeletion status. Another radiomics based approach was studied by Yu et al. [10] on IDH mutation prediction. Van der Voort et al. [11] extracted 78 MR image features and applied support vector machine (SVM) on them together with age and sex information for 1p/19q status prediction. Zhang et al. [12] also used SVM based approach for prediction of IDH mutation. These methods are based on conventional machine learning methods without automatic feature learning from brain MRIs.

The recent development of deep learning methods has drawn much attention for brain image analysis [13,14,15]. These methods may provide solutions for predicting molecular subtype gliomas by automatic feature learning. Matsui et al. [6] proposed a residual network-based model using multiple scans from MRI, positron emission tomography (PET) and computed tomography (CT) along with different characteristics of patients as numeric data for predicting three categories of molecular subtype. Liang et al. [16] applied 3D DensNets using multi-modal MRIs for IDH mutation prediction. However, deep models often require large amount of annotated data, and the dimension of features is rather high due to the complexity of the high dimensional input data (e.g., 3D medical images). Although convolutional neural networks (CNNs) are frequently used for characterizing visual objects in computer vision, deep autoencoder (AE) is often adopted as well. Deep AE is another type of deep learning method for characterizing images, however, the principle of AEs is different from that of CNNs and is based on applying codebooks (encoder and decoder) and generating codes. Additional advantages of AEs can include, e.g., noise robustness and feature reduction (depending on codebook size). It can also be used for both supervised and unsupervised learning. Further, the trained encoder part of the convolutional AE (CAE) [17,18] could also be used as a CNN. Such a setting can be benefited by first applying pre-training using a CAE for learning the manifold of dataset in a self-supervised way, followed by further refining the learning of network by learning complex features through supervised refinement. Such an approach has shown an improved generalization performance as compared to training the networks from the scratch with a small dataset [19]. Observing these advantages of CAEs along with considering our application, where MRI data could be noisy, we decided to select CAEs as the method for deep learning of brain tumor features in our study.

One practical challenging issue of using clinical dataset for glioma subtype classification is that the available medical datasets are often rather small, as they are usually collected by a local hospital from a region of a country. It might be desirable to learn a model on a specific subset of data [20,21]. For example, a hospital may require a model to be deployed that might perform well only on the hospital’s patient population. However, using the limited data from a single hospital might not be well enough to learn an accurate model causing generalization problem and achievements made for one hospital is not true progress unless it can be disseminated to other settings as well. Recently, a new data augmentation technique and its variations have gained popularity, known as Generative Adversarial Networks [22]. The GAN frameworks have been used in various medical imaging applications [23,24]. Most studies have proposed image-to-image translation such as label-to-segmentation [23], segmentation-to-image [25] or cross-modality translation [24,26]. Inspired by the above, we have decided to investigate deep convolutional GAN for augmenting synthetic training data in addition to existing data to improve the generalization performance (i.e., on the test set).

Another very challenging issue encountered in the real clinical application is that when there are many small glioma datasets, simply merging them into one dataset would not lead to significant increase of the generalization performance (i.e., on the test set) of the classifier. This is probably due to many reasons, for example, the MR image settings depend on the applied magnetic field, the radio pulse sequence frequency, the algorithm that the device follows for image reconstruction and so on. Hence the scanner dependent distribution of MRIs from different devices under different settings therefore tend to be creating feature mismatch [27]. This mismatch has been overcome majorly by two methods: global histogram-matching methods [28,29] and joint histogram registration method [30,31]. However, these methods work on paired-MRIs from source to target domains which are difficult and expensive to obtain. Recently, domain adaption using deep learning techniques gain much attention in the areas of computer vision [27,32]. However, for medical image datasets, especially for glioma datasets, such studies are in their infant stage. A particular challenging issue is whether one may obtain an effective domain mapping method that is able to map between MRI datasets, in the meantime, retaining the molecular-subtype information after the mapping.

Our work is mainly motivated by the following issues: molecular marker-information in low grade gliomas (LGGs) are rather recently integrated and most datasets are small; the mismatches that arise when multiple datasets from different sources are combined together to enlarge the data size. Considering these challenges, our work is focused to propose a robust method by domain mapping to overcome the scanner dependent mismatches that preserves the molecular structural originality of gliomas. In this paper, we propose a novel approach based on CycleGAN [33] and multistream convolutional autoencoder framework [34] as a classifier. Although CycleGAN has been applied for non-medical applications [33] and cross-modality translation of MRIs [26], to the best of our knowledge this is the first work used for domain mapping that retains molecular-subtype information in low grade gliomas. Moreover, the data used in this work is raw clinical data for the prediction of 1p/19q codeletion and IDH genotype without annotations (tumor segmentation masks) obtained from multiple hospitals. The main contributions of this paper include:

- Propose a domain adaptation method based on unpaired-CycleGAN that maps several small datasets into a common one while preserving molecular biomarker information of brain tumors.
- Propose to enlarge the training dataset after mapping, using deep convolutional GAN (DCGAN) to produce augmented multi-modality MRIs (T1 weighted with contrast enhanced (T1ce), FLAIR).
- to tackle the crucial and time consuming task of accurate tumor segmentation which needs time and anatomical expertise to put soft tissue boundaries, a rectangular tight bounding box is used instead.
- Propose a multi-stream convolutional autoencoders (CAEs) and feature fusion scheme for deep learning of molecular-level information from MRIs in the mapped domain, where pre-training is applied on GAN augmented MRIs, while refined training is applied on MRIs from mapped domain.
- Extensive empirical tests and performance evaluation on the effectiveness of the proposed scheme and comparison with some state-of-the-art methods.

It is worth mentioning that although a part of this work has been presented in [34] which was based on classification of low and high grade gliomas, however, this paper applies to classify molecular subtypes in LGGs instead of tumor grading. Furthermore, this paper differs in terms of: mapping multi-source data to a common domain; dealing with the clinical MRIs that are not uniform in size in all 3D directions; avoiding laborious task of defining soft tissue boundaries; and lastly, including empirical tests and evaluation on two clinical datasets from different hospitals.

## 2. Overview of the Proposed Method

We propose a novel approach based on unpaired-CycleGAN to overcome the scanner dependent domain mismatches while preserving the subtitle molecular-biomarker information of MRI data. The basic idea is to overcome the problem of LGG MRI data scarcity and make the small raw clinical data usable from multiple institutions for improved performance of subtype glioma classification, which consists of: (a) using unpaired-CycleGAN to map source domain MRIs (FLAIR, T1ce) to target domain MRIs. (b) The combined MRI data are still small in size because of (i) still less number of subjects, (ii) poor resolution of 3D MRI at sagittal and coronal views, (iii) large class imbalance in IDH genotype. Therefore, deep convolutional GAN (DCGAN) is used to augment synthetic MRIs across different modalities to enlarge the training data. The fake generated MRIs cover more tumor statistics that offer more robustness to its distribution although they look similar to real MRIs visually [22]. (c) Extracting high-level glioma features through applying 2-streams of convolutional autoencoders (CAEs) from multi-modality MRIs (T1ce, FLAIR) that is followed by information fusion with 2-stage training strategy. The augmented MRIs are used for pre-training to capture the glioma features while the real MRIs are used for refined training.

**Figure 1:**
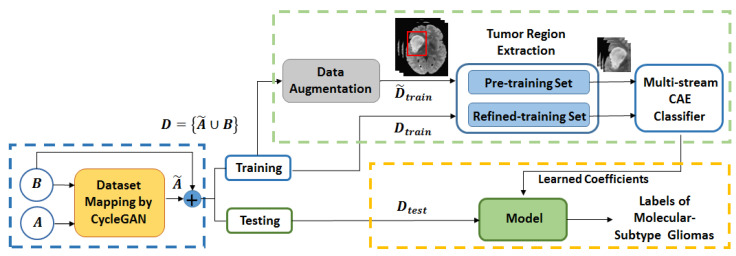
The pipeline of the proposed method. Blue dash box: domain mapping of dataset; Green dash box: feature learning and training process; Yellow dash box: testing process.

Figure 1 shows the block diagram of the proposed scheme for LGG-subtype glioma prediction based on clinical MRI data from two hospitals. Input 2D images from multi-modality MRIs (T1-contrast enhanced (T1ce), FLAIR) are fed into CycleGAN for mapping from source domain *A* to target domain *B* to generate mapped 2D images $\tilde{A}$ for each modality. These mapped data are added to the target domain to obtain total data *D*. To further enlarge the size of training data $D_{train}$ for each modality, image augmentation is done by employing deep convolution GAN (DCGAN) [35]. As the datasets have no tumor masks, the tumor regions are extracted by fixing a tight rectangular bounding box around ROI of images. These images with only tumor regions are used in a two step training strategy by 2-streams of convolutional autoencoder (CAE) [34]. During pre-training, phase features are learned from augmented images ${\tilde{D}}_{train}$ (T1ce-MRI and FLAIR-MRI). In refined training stage, features are fine tuned from $D_{train}$ MRIs in two streams which are further followed by feature fusion and two fully connected layers for prediction. Once the model is trained (green dashed box in Figure 1), the prediction is made on test data $D_{test}$ (yellow dashed box). In the remaining of this section, we shall give further details to explain the block from the blue dashed box (CycleGAN for domain mapping in Section 2.1) and the green dashed box (data augmentation in Section 2.2 and multi-stream CAE classifier in Section 2.3) from Figure 1 with their corresponding architectures. Section 3 describes the experimental setup, obtained results and comparison with the existing methods. Finally, in Section 4 conclusions are drawn from discussion.

### 2.1. Unpaired Cyclegan for Domain Mapping

Among many Generative Adversarial Network (GAN) models for image-to-image transformation, we selected CycleGAN [33] for mapping realistic images from source domain to the target domain aiming to increase the data size by combining the datasets from multiple sources. A conventional GAN consists of two-sub networks: a generator and a discriminator. A generator learns to produce fake image distribution similar to the real image distribution while discriminator learns to distinguish between both distributions. Both the networks are trained simultaneously to reach an optimal solution by minimizing the adversarial loss. In contrast, a CycleGAN uses two inputs in two streams, different from GAN that consists of one stream of input. In addition to adversarial loss, CycleGAN aims to also minimize the cycle-consistency losses.

#### 2.1.1. Formulation of the Unpaired Cyclegan

The idea is to learn the two mappings between the two unpaired sources of data *A* and *B* respectively.

**Figure 2:**
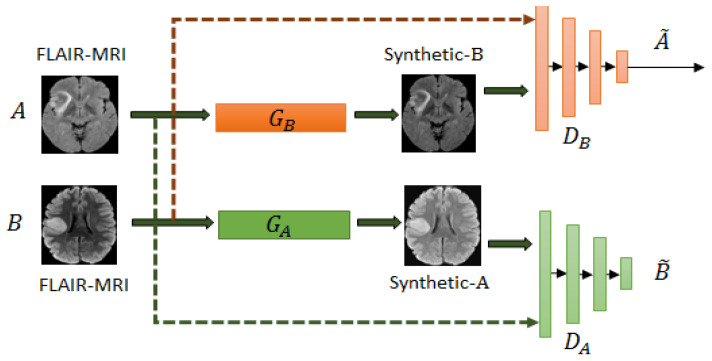
Example of unpaired Cycle Generative Adversarial Network (CycleGAN) used for mapping images from domain *A* to domain *B* for FLAIR-MRIs. The generators are $G_{A}$ and $G_{B}$ and the discriminators are $D_{A}$ and $D_{B}$.

As shown in Figure 2, MRIs (FLAIR, T1ce) from two datasets are inputs to their corresponding generators $G_{B}$ and $G_{A}$. The two output discriminators $D_{B}$ and $D_{A}$ are to compare the corresponding real images from the synthetic ones. The objective of the unpaired CycleGAN is given as:

(1) $$\begin{array}{c} L\left(G_{A},G_{B},D_{A},D_{B}\right)=L_{GAN}\left(G_{B},D_{B},A,B\right)+\\ L_{GAN}\left(G_{A},D_{A},B,A\right)+\lambda L_{cyc}\left(G_{A},G_{B}\right) \end{array}$$

where

(2) $$\begin{array}{c} L_{GAN}\left(G_{B},D_{B},A,B\right)=E_{b∼p_{data}\left(b\right)}\left[{\left(D_{B}\left(b\right)-1\right)}^{2}\right]\\ +E_{a∼p_{data}\left(a\right)}\left[D_{B}^{2}\left(G_{B}\left(a\right)\right)\right] \end{array}$$

Similarly, $L_{GAN}\left(G_{A},D_{A},B,A\right)$ can be defined as in (2),

(3) $$\begin{array}{c} L_{cyc}\left(G_{B},G_{A}\right)=E_{a∼p_{data}\left(a\right)}[∥G_{A}\left(G_{B}\left(a\right)\right)-a{∥}_{1}]\\ +E_{b∼p_{data}\left(b\right)}[∥G_{B}\left(G_{A}\left(b\right)\right)-b{∥}_{1}] \end{array}$$

We denote the data distribution as *a*∼$p_{data}\left(a\right)$ and *b*∼$p_{data}\left(b\right)$ respectively given the training samples ${\left\{a_{i}\right\}}_{i=1}^{N}$ from domain *A* and ${\left\{b_{j}\right\}}_{j=1}^{M}$ from domain *B*. $G_{B}$ is the generator that takes {a} an input dataset and generates the mapped dataset $\left\{\tilde{a}\right\}$, $D_{B}$ is the discriminator and aims to discriminate between the real {b} and augmented $\left\{\tilde{a}\right\}$ images. For estimating cycle-consistency and reversible mappings between the two domains, it uses explicit reconstruction error to ensure the cycle-consistency and to reduce $L_{cyc}$ in (3), where λ is the regularization parameter. For stable training, the least square loss is used in $L_{GAN}$ compared to the conventional negative log likelihood. The optimized generator and discriminator are obtained by training on the total loss in (1):

(4) $${G_{A}}^{*},{G_{B}}^{*}=\arg\min_{G_{A},G_{B}}\max_{D_{A},D_{B}}L\left(G_{A},G_{B},D_{A},D_{B}\right)$$

The unpaired CycleGAN learns to map realistic MRIs from the source domain *A* to the target domain *B* without any correspondence from small datasets at both ends therefore it is named as unpaired. The mapped domain $\tilde{A}$ is now in the desired domain *B* that has overcome the scanner dependent differences and matches the sample distribution of target domain *B* preserving the tumor characteristics on molecular level. The total data $D=\{\tilde{A}\cup B\}$ are used for further processing in the pipeline as shown in Figure 1.

#### 2.1.2. Architecture of Unpaired Cyclegan

**Figure 3:**
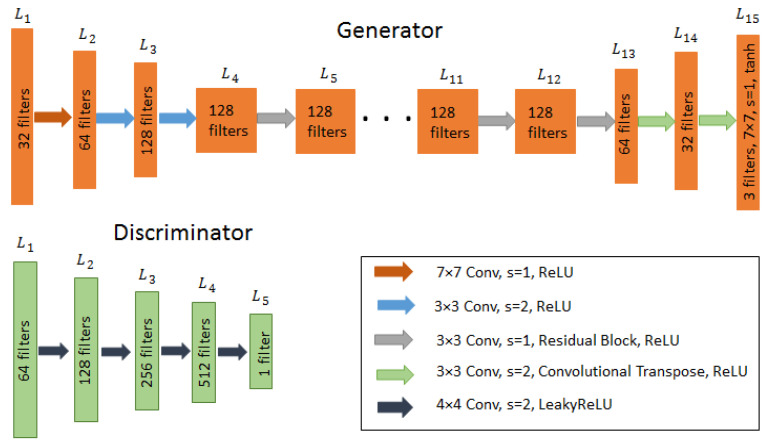
Architecture of the generator and discriminator of unpaired CycleGAN. Conv: 2D Convolutional filter, s: Stride, ReLU: Rectifier linear unit.

The architectures of both the discriminators and the generators are shown in Figure 3.

The input image to the generator is fed to a series of three convolutional layers which shrink the representation with increasing number of channels. The numbers of filters are set to 32, 64 and 128, respectively. It is then followed by a series of 9 residual blocks each set with 128 filters. The stream is further expanded using transpose convolutional layers to enlarge the representation for generating the final image. The numbers of filters selected are 64, 32 and 3, respectively. Each layer is followed by an instance normalization and ReLU as the activation function except Tanh in the last layer for reconstruction. This setup has been taken from [33] and adjusted accordingly for this specific application. For the discriminator, Markovian discriminator (PatchGAN) [36] is used to distinguish whether the image patches are real or fake. It has fewer parameters, less computational cost and can handle arbitrary image size compare to the full-image discriminator. For its stable and better training results, the least square loss function is used rather than the conventional negative log likelihood function. The discriminator consists of five layers with number of filters set to 64, 128, 256, 512 and 1, respectively. The first four convolutional layers have filter size 4 × 4 and LeakyReLU as activation function to introduce a small positive gradient when a neuron is not active. The last layer ends with a sigmoid function.

### 2.2. Data Augmentation by Deep Convolutional GAN

This part explains the data augmentation block in the pipeline from Figure 1 to generate augmented synthetic data ${\tilde{D}}_{train}$. In medical imaging, insufficient training dataset is partially resolved by slicing the 3D-MRIs to 2D slices with the maximum number covering tumor regions. Usually if data has enough resolution in all directional views, 2D slices are extracted from all directions of 3D volume (e.g., axial, coronal and sagittal). However, this strategy helps to some extent to increase diversity in training set and prevents the model from over-fitting. Since, the size of the datasets *A* and *B* are quite small which is still not sufficient to train a good predictive model. In this regard, we have used deep convolutional GAN (DCGAN) [35] for enlarging the training data size by generating augmented images for both modalities T1ce-MRIs and FLAIR-MRIs. Although the CycleGAN generated data are also considered as synthetic but because it preserves the anatomy of brain image from molecular level of tumor to the whole brain image unlike DCGAN, we call it here as mapped data. While the augmented distribution of data from DCGAN presents some differences, for instance; size of tumor, tumor location and introduce other structural differences. A detail description of the architecture is given in Table 1.

Unlike CycleGAN from Section 2.1 which accepts input as an image, here, the generator *G* learns a mapping from an input vector *z* (typically from a uniform distribution $p_{z}$) and maps to an image *y* in the target domain $p_{g}$. While discriminator *D* learns to distinguish between the true images *y* and the fake images G(y). While training, both *G* and *D* learn simultaneously where *G* aims to generate images with high probability to achieve the goal $p_{g}=p_{data}$ and look more real. Conversely, *D* learns aiming to discriminate the fake and true images. This is obtained by optimizing the given adversarial loss function in Equation (5):

(5) $$L_{GAN}\left(G,D\right)=E_{y∼p_{data}}logD\left(y\right)+E_{z∼p_{z}}log\left(1-D\left(G\left(z\right)\right)\right)$$

where *G* tries to minimize the loss function $L_{GAN}$ for images with *y*≁$p_{data}$ and *D* tries to maximize $L_{GAN}$ for images with *y*∼$p_{data}$ simultaneously. The aim is that *G* learns to produce more realistic augmented images that *D* might not differentiate from the real ones. For each MRI-modality, DCGAN is trained separately to synthesize the augmented images from the corresponding modality. A vector of 100 random samples drawn from a uniform distribution is given to the generator network as input to generate the augmented MR images and the discriminator compares the original and augmented images to output a decision: real or fake?

### 2.3. Review of Multi-Stream Convolutional Autoencoder and Feature Fusion

**Figure 4:**
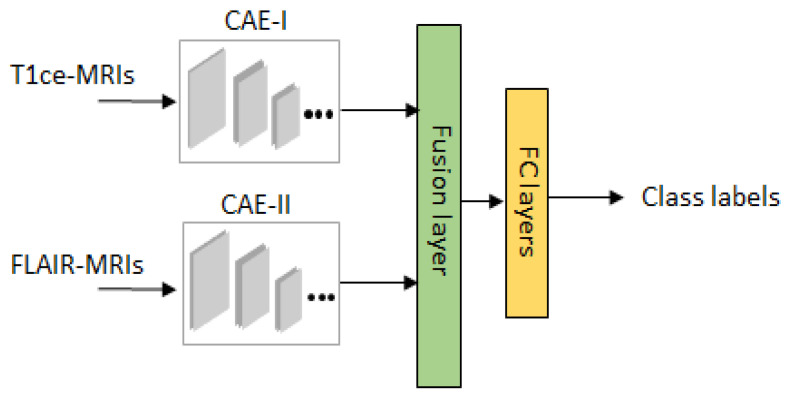
2-stream convolutional autoencoder (CAE)-based classifier for LGG-subtype classification.

For the sake of convenience to the readers, a brief overview of the classifier is given in Figure 4. After overcoming the possible mismatches between the two domains *A* and *B*, we have obtained data *D* in total. Moreover, both the datasets are available without the tumor masks so to allow the network to focus on learning the tumor characteristics, we have fixed a rectangular tight bounding box on the ROI (region of interest) of each image. This step is further proceeded with a two-stage training strategy based on our previous work on Multistream Convolutional Auoteoncder [25] as a classifier. By doing so, a noticeable performance is obtained from our empirical test results. A detailed architecture of one stream of classifier is described in Table 2. For the 2 modalities of MRIs, we train 2 convolutional autoencoders denoted as CAE-I and CAE-II. In each CAE, the encoder part consists of 6 convolutional layers for extracting high dimensional feature maps followed by the decoder with 5 convolutional layers for reconstruction. Since, this overcomplete representation gives the CAE possibility to learn the identity function. To prevent over representation, max-pooling is used to enforce the learning of plausible features.

We use two stage training strategy for our classifier network. In pre-training stage, both streams are unsupervisedly trained on GAN augmented data ${\tilde{D}}_{train}$ with the corresponding MRI modalities. The aim of this training phase is allowing the encoders learn generic features from augmented data ${\tilde{D}}_{train}$. In refine training stage, features learned by encoder layers from 2 streams are proceeded further by feature fusion for prediction where it has access to the data $D_{train}$ and the class labels. For the refinement of fused features and compact representation, aggregation and bilinear layers are used on fusion layers [37]. Let $f_{1}$ and $f_{2}$ denote the features from the last encoder layers of size h×w×c, where *h* is the height, *w* is the width and *c* shows the number of channels. The aggregated feature vector is obtained by element-wise multiplication as $f=f_{1}⊙f_{2}$ and hence the spatial relationship of features from both streams are maintained. The bilinear feature layer captures the interaction of features with each other at spatial locations by computing $H=f^{T}f$, where H is the final refinement map. Finally, fully connected layers are introduced each with 256 number of neurons with random initialization and dropout regularization. Then, a softmax layer is added that determines the class labels. This way of two stage training has been seen effective in learning generic features and fast convergence.

## 3. Experimental Results

### 3.1. Setup, Datasets, Metrics

#### 3.1.1. Setup

Implementation of our network was done using KERAS library [38] with Tensor Flow backend on a workstation with Intel-i7 3.40 GHz CPU, 48 G RAM and an NVIDIA Titan Xp 12 GB GPU. By tuning the network carefully through experiments, hyperparameters of CycleGAN were selected on an average of 150 epochs. The size of the mapped generated images was selected as 128*128. The learning rate was set to $2.0\times 10^{-4}$ that was linearly decayed after 100 epochs with *Adam* optimizer. For DCGAN network, again Adam optimizer was used but with a learning rate of α=0.002 and a binary crossentropy loss function. The training of GAN was continued until the output probability of discriminator approached to 0.5 called the Nash Equilibrium point. The mini batch size was set to 64. Finally for training the classifier, in pre-training stage of each stream of CAE, *Adam* optimizer with mean square error loss function, learning rate of α=0.002 and mini batch size of 16 were used for 200 epochs. The performance was evaluated by the loss vs. epochs curve. We used L2-norm regularization with the parameter value of $1.0\times 10^{-4}$ for convolutional layers of each stream of CAE. In the refined-training stage, the categorical cross-entropy was used as a loss function for evaluating the final performance. Here, we adapted early stopping strategy when the best validation performance was achieved. The random dropout rate was set to 0.5 for two fully connected layers. Simple data augmentations such as horizontal flipping and random rotation (maximum at $10^{\circ }$) were used as well during this real time training.

#### 3.1.2. Datasets

The datasets used in the evaluation are provided by two different hospitals for patients with known 1p/19q codeletion/non-codeletion and IDH mutation/wild-type status: USA dataset from University of California San Fransisco and France dataset from Department of Neurosurgery, University of Paris, GHU Paris, Sainte-Anne Hospital. Note that the data are unpaired which means that both the data sources are from two different institutions having no subject in common. Unlike other MRI open datasets, the patient’s tumor mask annotations and other demographic characteristics are not available for both datasets. Based on the availability of modalities, class labels and quality of scan, 82 subjects were selected out of 87 from France dataset and 79 subjects out of 95 were used from USA dataset. The data consists of 3D brain volume but we have used slices from only axial views as the number of slices were not sufficient in the coronal and sagittal views in majority of subjects. The summary of the datasets is given in Table 3a.

**Partition of Dataset for Multiple Runs:** Since deep learning requires heavy computation, we adopted the commonly used approach by averaging several test runs as the performance index (rather than cross-validation in conventional machine learning). This is done as follows: for each new run, a new partition is performed to split the dataset into subsets of training (60%), validation (20%) and testing (20%), where strictly patient-separated partition is applied (i.e., MRI slices from each patient would only be used in one of the subsets). Then, the training process is repeated, i.e., applying GAN data augmentation on the new training subset, followed by pre-training of GAN augmented data and refined-training of multi-stream CAE with mapped MRIs (using the same hyperparameters and network architecture in all runs). After that, the testing process is applied by using data from the new test subset for feature extraction and classification. The test performance obtained from such 5 runs are then averaged for the final performance evaluation.

Based on the confirmed histological identification of subjects as LGGs, we considered two case studies as shown in Table 3b. Two modalities of images, T1ce-MRI and FLAIR-MRI were used in the tests.

**Case-A:** This case was applied for classifying subtype-LGG 1p/19q codeletion and non-codeletion. From Table 3b, one can see that 77 patients are 1p/19q codeleted and 84 patients are non-codeleted. Observing that the tumor size varies in each subject, 10 slices for each glioma were extracted from each 3D scan for training the multi-stream CAE classifier.

**Case-B:** This case was designed for classifying IDH genotype. From Table 3b, one can see that 137 patients are labeled as IDH mutated and 24 patients as IDH wild-type. Unlike Case-A, the same datasets have large class imbalance for IDH genotype. Therefore, 3 time slices have been extracted for patients from IDH wild-type class.

#### 3.1.3. Metrics for Performance Evaluation

To evaluate the performance of diffuse LGG-subtype classification, objective metrics were used based on the following four kinds of samples.

True positive (TP): the 1p/19q codeletion/IDH mutation gliomas, and were correctly classified as 1p/19q codeltion/IDH mutation.
False positive (FP): the 1p/19q non-codeletion/IDH wild-type gliomas, but were incorrectly classified as 1p/19q codeltion/IDH mutation.
True negative (TN): the 1p/19q non-codeletion/IDH wild-type gliomas, and were correctly classified as 1p/19q non-codeltion/IDH wild-type.
False negative (FN): the 1p/19q codeletion/IDH mutation gliomas, but were incorrectly classified as 1p/19q non-codeletion/IDH wild-type.

The metrics computed were defined as accuracy, precision, recall/sensitivity and F1-score given as follows:

$$\mathrm{Accuracy}=\frac{\mathrm{TP}\;+\;\mathrm{TN}}{\mathrm{TP}\;+\;\mathrm{FP}\;+\;\mathrm{TN}\;+\;\mathrm{FN}},\;\mathrm{Precision}=\frac{\mathrm{TP}}{\mathrm{TP}\;+\;\mathrm{FP}}$$

$$\mathrm{Recall}=\frac{\mathrm{TP}}{\mathrm{TP}\;+\;\mathrm{FN}},\;F1-\mathrm{score}=2\times \frac{\left(\mathrm{Recall}\times \mathrm{Precision}\right)}{\mathrm{Recall}\;+\;\mathrm{Precision}}$$

### 3.2. Pre-Processing and Tumor Bounding Box

#### 3.2.1. Pre-Processing

This step has an impact on the performance. The clinical 3D volume scans in both the datasets were unregistered. Thus, the anatomical images from FLAIR and T1ce scans were registered to 1 mm MNI space template. In addition to this, the bias field correction and skull-stripping steps were performed using FSL [39] and ANTs [40] tools. To save computation, slices were rescaled to a 128×128 size and then normalized to range [0,1]. For training CycleGAN, we used all the axial cross sections that contained artifact-free brain tissue from each subject. While training DCGAN for generating augmented images, all images with tumor regions were selected. However, for refined training only 10 slices with tumor parts were used.

#### 3.2.2. Tumor Bounding Box

**Figure 5:**
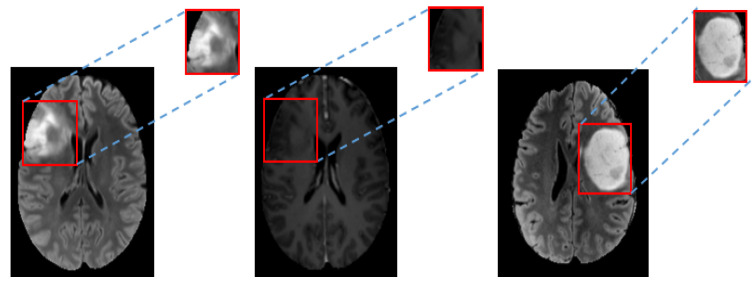
Example of allocated tumor regions by rectangular bounding boxes for tumor feature learning. Left to Right: FLAIR, T1ce and FLAIR.

A tight bounding box of rectangular shape was used by allocating the tumor region on each image. Images of tumor regions were then used as the input for more efficient tumor feature learning. Figure 5 shows an example of tumor regions used for feature learning. After that, all tumor regions were normalized to 64 × 64 pixels.

### 3.3. Results and Discussions

#### 3.3.1. Performance Evaluation on the Impact of Individual Parts

The purpose of this study is to establish a framework that can enable data to be used from multiple domains for improved performance. To investigate the effectiveness of our approach on each case study, first we had to choose which dataset should be mapped to the other. In this regard, we did a primarily test using multi-stream CAE classifier [34] on the USA and France datasets separately and then combined them without applying domain mapping. Then we compared the performance with combined dataset obtained from after domain mapping. Furthermore, we also examined the effect of using GAN augmented data for pre-training. Finally we applied all these methods in the proposed scheme and evaluated the overall performance of the pipeline.

**Figure 6:**
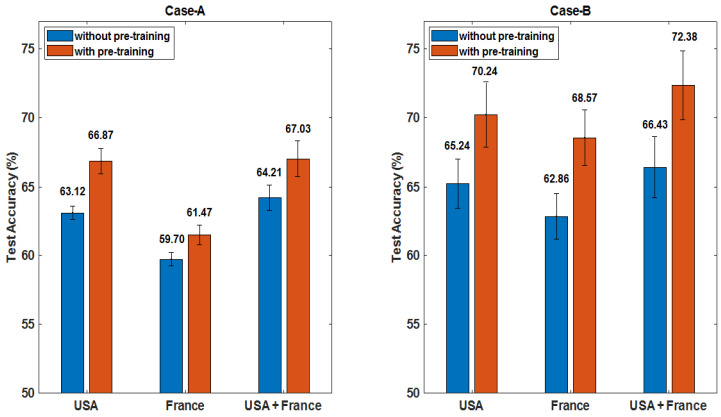
Comparison of test performance on individual dataset and on combined dataset (without domain mapping). Further the effect of using GAN augmented data for pre-training is also examined (red bars) as compared with those without using GAN augmented data (blue bars). **Left:** Case-A: pre-training effect on classification of 1p/19q codeletion/non-codeletion has shown improvement by about 3%. **Right:** Case-B: pre-training effect on classification of IDH mutation/wild-type has shown improvement by about 5%.

**Test Performance Comparison on Data without Domain Mapping on the Effect of Pre-training:**Figure 6 shows the test performance from using individual dataset (USA dataset, France dataset) and the simply combined two datasets (USA+ France). Noting in this set of experiments, we also compared results from with and without using GAN augmented data for pre-training (shown in blue and red bars in Figure 6).

Observing the results from Case-A in Figure 6 (Left), it is found that the USA dataset gives better prediction of 66.87% on test data as compared to the France dataset which is 61.47%. After, when data were combined without mapping, the performance increased slightly up to 67.03% but not to a noticeable extent compared to the size of second dataset added, possibly because of the domain mismatches. Note that the pre-training effect increased the performance by nearly 3%.

Observing the results from Case-B in Figure 6 (Right), one can see a similar trend of behavior but additionally improved performance is observed for IDH1 mutation/IDH1 wild-type classification. Again for this case, USA dataset gave better test result which is 70.24% and increased up to 72.38% when combined with France dataset. Note that the pre-training effect increased the performance efficiently by 5%. In this experiment, the reported test results were averaged over 3 runs for both cases. Hence, USA dataset was set as the target domain and France dataset as the source domain in training the unpaired CycleGAN.

**Figure 7:**
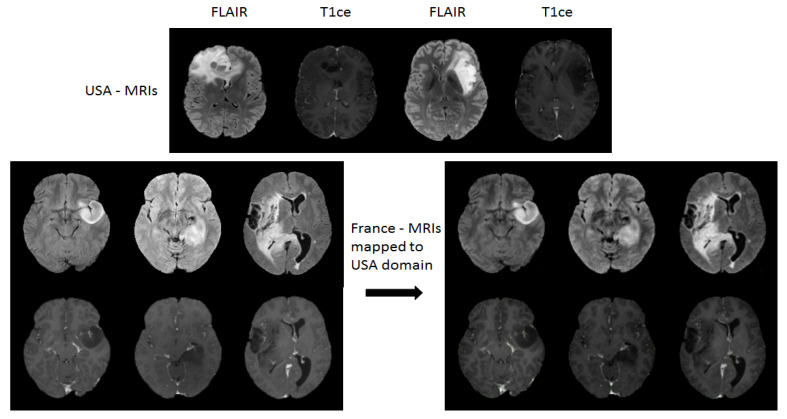
Visual inspection of image slices before and after the domain mappings. **Top:** Examples of FLAIR-MRIs and T1ce-MRIs from USA dataset. **Bottom left:** 3 original 2D slices of FLAIR-MRIs (1st row) and T1ce-MRIs (2nd row) from France dataset. **Bottom right:** Domain mapped 2D slices of FLAIR-MRIs (1st row) and slices from T1ce-MRIs (2nd row) from France to USA domain.

**Effect of Domain Mapping:** Domain adaption was then applied by mapping the French dataset to the USA data domain. Figure 7 shows an example of visual effect of images before and after the mapping. The domain mapped dataset has also been visually inspected by medical doctors, where domain mapped French dataset showed consistently more similar distribution as that of USA dataset. Moreover, the impact of domain mapping on yielding improved test performance has been discussed in detail in Section 3.3.2.

**Figure 8:**
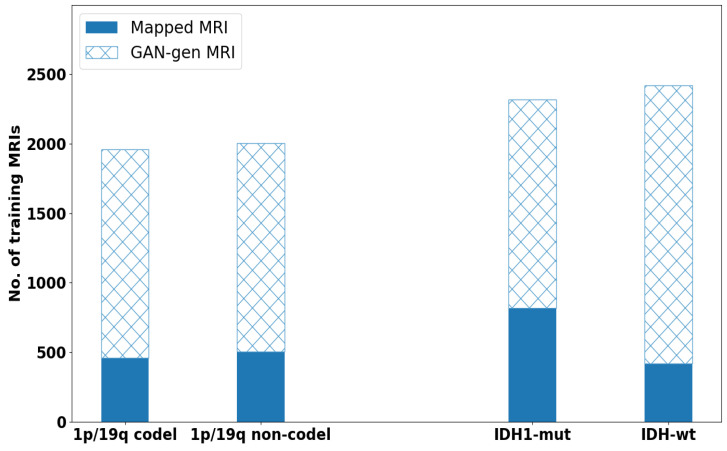
Total number of data (GAN augmented + Mapped MRI) for one modality of training set, i.e., 60% in both case studies.

**Impact of GAN Augmented Data:** Two issues were studied here: One is how big the size of augmented data, another is whether one should use augmented data for pre-training or mixed training with the mapped measured data. To evaluate the quality of augmented images generated by DCGAN, a single stream of CAE was trained with FLAIR-MRIs for both molecular-subtype LGGs. After testing the pre-training and the mixed training approaches, we adopted a two-stage training strategy: pre-training on GAN augmented data, refined training on domain mapped data. We then tested on adding different sizes of augmented images on training set (60%) in both Case-A and Case-B studies. Figure 8 depicts the total size of data (augmented + mapped data) used for our evaluations in both cases.

**Figure 9:**
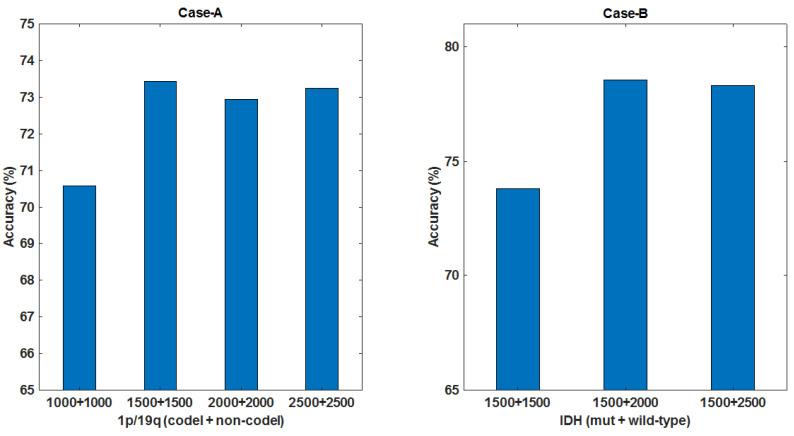
Test accuracy vs. number of augmented data used for pre-training. **Left:** Case-A: equal number of augmented images are used for each class. Noting in the horizontal axis, **(1500 + 1500)** is the augmented data size selected. **Right:** Case-B: more number of augmented images are added to the class with smaller set to balance the data and **(1500 + 2000)** is the selected augmented data size.

Considering Case-A, in which 2 classes are well balanced, we took equal number of augmented images for each class. Different size of augmented images were tested for pre-training. Observing Figure 9 (Left), the test results shows that the performance did not improve much after the size (1500 + 1500) of the augmented images for the 2 classes. This indicates the limit of diversity which augmented images offered during pre-training. A best of 73.44% test accuracy was obtained after refine training with 460 and 500 images for 1p/19q-codeletion and non-codeletion. The total data used for both classes are shown in Figure 8.

In Case-B, as the 2 classes were not well balanced, we took more augmented images for the class with smaller set. Observing Figure 9 (Right), test results show that increasing the augmented image size beyond (1500 + 2000) for 2 classes that gave 78.57% accuracy showed no any noticeable improvement. The accuracy was obtained after the refined training stage with 820 and 420 MRIs for IDH mutation and wild-type, respectively. The total data used are shown in Figure 8. In other sets of experiments, these selected sizes of augmented MRIs were used.

#### 3.3.2. Overall Performance of the Proposed Scheme

**Figure 10:**
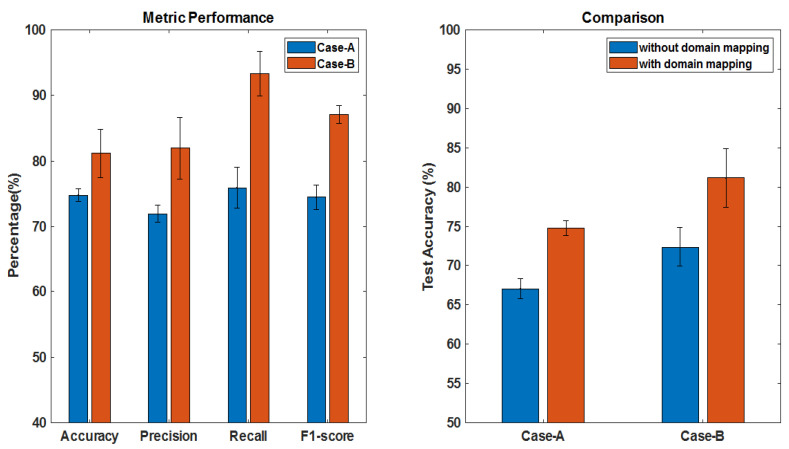
**Left:** Test performance of the proposed scheme (averaged over 5 runs) for both case studies. **Right:** Test performance of with/without domain mapping for both case studies.

In this set of experiments, we evaluated the overall performance of the proposed pipeline (see Figure 1) for both Case-A and Case-B. The final results from 2-stream feature fusion and CAE classifier on the combined dataset after mapping are shown in Table 4. Columns 3 and 4 show the test results from applying a single modality from mapped dataset, while column 5 shows the test accuracy from fusion of 2 modality information. Noting that the test accuracy on FLAIR-MRIs is better as compared to that on T1ce-MRIs in both case studies, probably due to better contrast of tumor regions against the background tissues. Observing the results from using 2 modality inputs, the features learned from both scans were combined through feature fusion layers which increased the prediction on test accuracy. In Table 4 (columns 6–8) and Figure 10 (Left), we also show the performance by applying 3 metrics: precision, recall and F1-score, for further evaluation purpose comprehensively. The results are reported on 5 runs where for each run the three sets of data (training, validation and test) were selected randomly.

**Figure 11:**
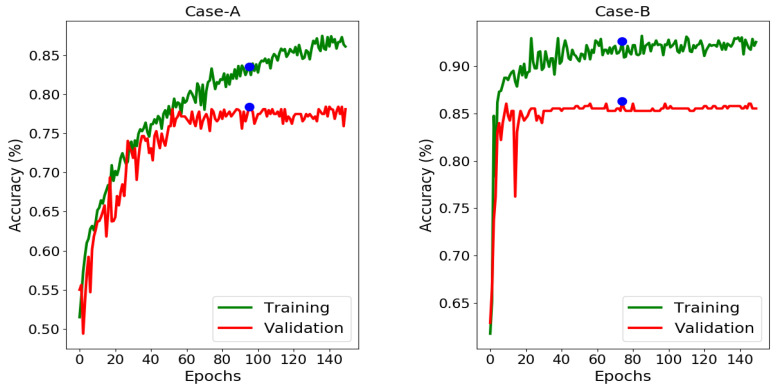
Training and validation performance as a function of epochs from the refined training (green) and validation (red) curves. Early stopping strategy was applied, where blue dot points to the epoch whose parameters were used for test set. **Left:** Case-A: the validation curve converged at epoch = 95 and then stabilizes. **Right:** Case-B: the validation curve converged smoothly at epoch = 74 and then stabilizes.

Observing the results of Case-A for the performance comparison of with/without domain mapping from Figure 10 (Right), the test accuracy reached to an average of 74.81% (improved by 7.78%) that shows a noticeable increase in the accuracy for prediction of 1p/19q codeletion. Observing the refine training curve in Figure 11 (Left), the validation accuracy (78.44%) was obtained at epoch = 95 with the training accuracy (83.54%) that gave the best test accuracy (76.09%). Precision (71.95%) indicates here that how many patients predicted as 1p/19q codeleted are actually codeleted. Recall (75.93%) indicates correctly predicted 1p/19q codeleted patients out of all codeleted ones. As class distribution is nearly equal, accuracy (74.81%) can be considered a better metric compared to its F1-score (74.50%).

Unlike the previous case, the class distribution in Case-B for IDH mutation and IDH wild-type is uneven. The test accuracy reached to 81.19%. Precision (81.96%) indicates here that how many patients predicted as IDH mutated are actually mutated. Recall (93.33%) indicates the correctly predicted IDH mutated patients out of all mutated ones which is an increased positive classification rate. In this case, F1-score proves to be a better metric for evaluation with an average value of 87.09% due to imbalance classes. Again, observing Figure 10 (Right) for performance comparison of with/without domain mapping, a noticeable increase in accuracy (81.19%, improved by 8.81%) was achieved. Noting the refine training curve from Figure 11 (Right), where best test accuracy (85.71%) was obtained at epoch = 74 with training accuracy (92.60%) and validation accuracy (86.25%).

A summary of the overall performance, where different metrics are shown in Figure 10 (Left) and the effect of domain mapping to the overall performance improvement in Figure 10 (Right), shows that the proposed scheme is effective, and could be a useful approach for further research on molecular subtypes prediction from MRIs.

### 3.4. Comparison with State-of-the-Art and Discussion

To further evaluate the proposed scheme, we compare our performance with several state-of-the-art results on prediction of 1p/19q deletion/non-codeletion and IDH1 mutation/wild-type. There exists some reported work on these molecular-subtype gliomas using open datasets [41,42], but we have mostly selected few ones for comparison that have used clinical datasets as shown in Table 5.

It is worth mentioning that these comparisons can only be used just as an indication because they were applied to different datasets with different scan types, MRI modalities and patient’s characteristics. For instance, Matsui et al. [6] used residual network-based deep network that required more modalities of data (FLAIR, T1ce, T1, T2), including PET and CT scans in addition to other side information of patients as numeric data. Zhou et al. [7] used hand-crafted features such as histograms, shape and texture from data that was collected from single institution combined with age information for a random forest classifier. Han et al. [5] used an analysis to generate radiomics signature by extracting 647 MRI-based features from T2-MRIs and side information of patients. Van der Voort et al. [11] used support vector machine classifier to extract features from T1 and T2-MRI along with age and sex information on 284 patients and validated results on 129 patients from TCIA. Yu et al. [10] used radiomics based approach on FLAIR-MRI data from single hospital. However, the method required segmented tumor masks, tumor characteristics (tumor location and volume) and other numeric data of patients. Zhang et al. [12] introduced a method on 103 patients from TCGA and TCIA data based on the availability of their quantitative texture, histogram features and scan type (T1, T1ce and T2, FLAIR) availability. All these methods were based mostly on using a single and relatively large dataset. Observing the last row for each case in Table 5, our proposed scheme indicates relatively good performance on using moderate data size of 161 patients with two MRI modalities obtained from two institutions, provided with no segmented tumor masks or other patient’s/tumor’s side information for assisting classification. This also supports that the performance is comparable to the state-of-the-art.

**Limitation and Future Work:** Despite the method is promising in domain mapping and molecular-subtype classification, some challenges remain before putting this tool into clinical usage. Further improvement is required to increase the test performance, for example, to make the network work more robustly, more datasets from multiple institutions should be added through domain adaptation to enlarge the training data. The imbalance between the classes needs to be mitigated by seeking more augmented data. As a future work, we can compare the strategy of bounding box with true tumor mask segmentation. Moreover, increasing the number of classes by combining IDH genotype and 1p/19q codeletion status into one classifier would also be desirable for the clinical usage.

## 4. Conclusions

Since the introduction of molecular-markers in LGGs was implemented recently in the WHO 2016 classification, the data availability is quite low. The proposed scheme has been tested to enlarge the clinical datasets from independent sources and to overcome the domain mismatches of the datasets for the prediction of molecular-subtypes for LGGs. The results obtained on the test dataset have shown a noticeable increase in the performance compared to when the dataset was not mapped (74.81%, improved by 7.78% on 1p/19q codeletion status) and (81.19%, improved by 8.81% on IDH mutation status). It shows that unpaired CycleGAN has maintained the subtitle-molecular information while mitigating the domain differences. The effect of pre-training and its effect with GAN augmented images has both resulted in increased generalization performance of multi-stream CAE classifier. In addition, instead of time consuming and laborious task of putting exact tumor boundary, the method of using bounding box around the tumor proved to be effective. Although test results obtained by the proposed scheme indicate promising performance compared to the state-of-the-art, but this comparison should be considered just as an indication because different methods have used different data size and scan types. Further, we discussed limitations of the method and some possible future work.

## Figures and Tables

**Table 1:** Deep convolutional GAN (DCGAN) architecture.

Layer	Filters	Output Size
Discriminator *D*:		
Conv-1 + stride 2 + BN + LeakyReLU(0.2)	5×5×128	32×32×128
Conv-2 + stride 2 + BN + LeakyReLU(0.2)	5×5×256	16×16×256
Conv-3 + stride 2 + BN + LeakyReLU(0.2)	5×5×512	8×8×512
Conv-4 + stride 2 + BN + LeakyReLU(0.2)	5×5×1024	4×4×1024
Dense + sigmoid	-	1
Generator *G*:		
Dense + ReLU + reshape	2,662,144	16×16×1024
ConvTranspose-1 + stride 2 + BN + ReLU	4×4×512	32×32×512
ConvTranspose-2 + stride 2 + BN + ReLU	4×4×256	64×64×256
ConvTranspose-3 + stride 2 + BN + ReLU	4×4×128	128×128×128
Conv-5 + Tanh	4×4×3	128×128×3

**Table 2:** Architecture of CAE for a single stream.

Layer	Filters	Output Size
Encoder layer:		
Conv-1 + BN + ReLU	3×3×64	64×64×64
Conv-2 + Maxpool + BN + ReLU	3×3×128	64×64×128
Conv-3 + Maxpool + BN + ReLU	3×3×128	32×32×128
Conv-4 + BN + ReLU	3×3×256	16×16×256
Conv-5 + Maxpool + BN + ReLU	3×3×256	8×8×512
Conv-6 + BN + ReLU	3×3×512	8×8×512
Decoder layer:		
Upsample + Conv-7 + BN + ReLU	3×3×256	16×16×256
Conv-8 + BN + ReLU	3×3×256	16×16×256
Upsample + Conv-9 + BN + ReLU	3×3×128	32×32×128
Upsample + Conv-10 + BN + ReLU	3×3×128	64×64×128
Conv-11 + BN + ReLU	3×3×1	64×64×1

**Table:** (**a**)

Dataset	#3D Scans in T1ce	#3D Scans in FLAIR	# of Patients Selected
USA	85	79	79
France	82	84	82

**Table:** (**b**)

**Case-A: 1p/19q Codeletion Information**				
	**USA Dataset**	**France Dataset**	**# Patients**	**# 2D Slices T1ce/FLAIR**
1p/19q codeletion	44	33	77	77×10=770
1p/19q non-codeletion	35	49	84	84×10=840
**Case-B: IDH genotype information**				
IDH mutation	68	69	137	137×10=1370
IDH wild-type	11	13	24	24×30=720

**Table 4:** Average test results of 2 datasets with domain mapping for Case-A and Case-B for 5 runs. The highest value obtained in each run is displayed in bold text.

**Case-A: 1p/19q Codeletion/Non-Codeletion**							
Run	Dataset	T1ce	FLAIR	2-Modality	2-Modality	2-Modality	2-Modality
		Acc. (%)	Acc.(%)	Acc. (%)	Precision (%)	Recall(%)	F1-Score(%)
1		69.37	72.19	75.16	70.67	**80.33**	75.19
2	USA	**70.63**	71.56	**76.09**	72.48	79.00	75.60
3	+	69.69	**73.44**	73.44	70.57	74.33	72.39
4	France	69.69	72.81	75.47	**74.07**	73.33	**77.00**
5		70.00	73.13	73.91	71.95	72.67	72.31
	Mean ± ∣σ∣	**69.87** ± 0.43	**72.63** ± 0.67	**74.81** ± 0.98	**71.95** ± 1.29	**75.93** ± 3.12	**74.50** ± 1.85
**Case-B: IDH mutation/wild-type**							
1		71.67	75.24	81.43	79.81	95.18	86.82
2	USA	73.33	**78.57**	**85.71**	86.21	92.59	**89.28**
3	+	69.05	74.76	78.57	76.47	**96.29**	85.24
4	France	**75.00**	71.90	75.71	78.66	95.56	86.28
5		73.81	72.62	84.52	**88.68**	87.03	87.85
	Mean ± ∣σ∣	**72.57** ± 2.06	**74.62** ± 2.34	**81.19** ± 3.70	**81.96** ± 4.67	**93.33** ± 3.39	**87.09** ± 1.38

**Table 5:** Comparison with some existing state-of-the-art performance. It is worth noting that different datasets with different data size mostly from one source, different modalities and scan types were applied, hence these methods can be used as an indication or reference for “good” performance reported so far.

Case Study	Method	# of Patients	Test Accuracy (%)
Case-A	Zhou [7]	281	71.60
	Han [5]	277	72.00
	Van der Voort [11]	413	72.30
	Matsui[6]	217	75.10
	**Proposed Scheme**	**161**	**74.81**
Case-B	Yu [10]	140	80.00
	Zhang [12]	103	80.00
	Matsui[6]	217	82.90
	**Proposed Scheme**	**161**	**81.19**
